# The Effectiveness and Tolerability of Glycopyrronium for Patients with Chronic Obstructive Pulmonary Disease in a Clinical Setting: GLARE-Taiwan

**DOI:** 10.3390/jcm11206210

**Published:** 2022-10-21

**Authors:** Wei-Chang Huang, Sheng-Hao Lin, Liang-Wen Hang, Ching-Hsiung Lin, Jeng-Yuan Hsu

**Affiliations:** 1 Division of Chest Medicine, Department of Internal Medicine, Taichung Veterans General Hospital, Taichung 407, Taiwan; 2 Department of Post-Baccalaureate Medicine, College of Medicine, National Chung Hsing University, Taichung 402, Taiwan; 3 Ph.D. Program in Translational Medicine, National Chung Hsing University, Taichung 402, Taiwan; 4 School of Medicine, Chung Shan Medical University, Taichung 402, Taiwan; 5 Department of Medical Technology, Jen-Teh Junior College of Medicine, Nursing and Management, Miaoli 350, Taiwan; 6 Division of Chest Medicine, Department of Internal Medicine, Changhua Christian Hospital, Changhua 500, Taiwan; 7 Institute of Genomics and Bioinformatics, National Chung Hsing University, Taichung 402, Taiwan; 8 Department of Recreation and Holistic Wellness, MingDao University, Changhua 523, Taiwan; 9 Sleep Medicine Center, Department of Pulmonary and Critical Care Medicine, China Medical University Hospital, Taichung 404, Taiwan; 10 School of Physical Therapy, Chung Shan Medical University, Taichung, 402, Taiwan

**Keywords:** chronic obstructive pulmonary disease, Clinical COPD Questionnaire, effectiveness, glycopyrronium, tolerability

## Abstract

Glycopyrronium (GLY) is a pharmacological maintenance treatment for chronic obstructive pulmonary disease (COPD). However, its effectiveness and tolerability for COPD patients in routine clinical practice have not been well-investigated. This study aimed to assess the effectiveness of GLY on health-related quality of life and its safety in patients with COPD in a routine clinical care setting. This multi-center, prospective, six-month observational study recruited patients diagnosed with COPD and treated with GLY at three medical centers in central Taiwan. The full analysis set (*n* = 102) had a significant improvement in the Clinical COPD Questionnaire total (mean ± SD = −0.39 ± 0.90, *p* = 0.002), symptoms (mean ± SD = −0.61 ± 0.90, *p* < 0.001) and mental state scores (mean ± SD = −0.54 ± 1.72, *p* = 0.021) but not the functional state score (mean ± SD = −0.10 ± 1.15, *p* = 0.529). During the observational period, 58 patients (52.73%) experienced adverse events; only one adverse event (dizziness) was suspected to be related to the study drug. Three patients (2.73%) discontinued the study and GLY treatment because of an adverse event. One patient (0.91%) died during the study period because of a cerebral infarction, which was judged to be not associated with GLY treatment. In conclusion, GLY could be effective in improving the health status and is safe for patients with COPD in a real-life setting.

## 1. Introduction

Chronic obstructive pulmonary disease (COPD), a major cause of morbidity and mortality worldwide, is characterized by persistent pulmonary symptoms and airflow limitation due to airway and alveolar abnormalities [1]. These abnormalities commonly arise from significant exposure to noxious particles or gases [1]. As the disease progresses, the patient’s quality of life deteriorates with decreasing independence in daily activities and an increasing symptom burden [2]. To date, there is no cure for COPD, and patient management is focused on managing symptoms, improving the patient’s quality of life, and reducing future risks of exacerbation and mortality [1].

Despite the availability of several treatment options, inhaled bronchodilators remain the cornerstone of symptom control, improved exercise tolerance and quality of life, and reduced future risks of exacerbation for patients with COPD [1]. Long-acting muscarinic antagonists (LAMAs) are recommended first-line inhaled bronchodilators and used as monotherapies or in combination with other agents [1]. For this reason, they are increasingly prescribed for patients with COPD [1,3]. Inhaled glycopyrronium (GLY) is an LAMA that inhibits the bronchoconstriction effects of acetylcholine on M3 muscarinic receptors expressed in airway smooth muscles with faster dissociation from M2 muscarinic receptors. This prolongs the duration of the bronchodilation effect of GLY, thus being approved as a once-daily maintenance bronchodilator for the treatment of patients with COPD in many countries [4]. The GLOW trials (glycopyrronium bromide in COPD airways clinical study) showed that a once-daily treatment with GLY facilitated rapid and long-lasting improvements in the forced expiratory volume in one second. Furthermore, it improved patients’ health-related quality of life and exercise tolerance, reduced the risk of exacerbations and use of rescue medications, and exhibited a similarly safe and tolerable profile as compared to placebo or tiotropium [5,6,7,8]. Also, it has been found that GLY has a faster onset of action and greater bronchodilation effect within the first four hours after the first dose on day one of treatment [7]. Taken together, GLY is a bronchodilator agent to be considered primarily for COPD treatment.

Recent advancements in COPD treatment are aimed at improving not only the standard parameters of lung function but also patient-reported outcomes such as symptom severity, the extent of disability, and health-related quality of life [9]. These variables are key measurements to assess COPD treatment efficacy. With regard to health status, the Global Initiative for Chronic Obstructive Lung Disease committee suggests the COPD Assessment Test and Clinical COPD Questionnaire (CCQ), which are easy-to-administer, patient-reported questionnaires of supported validity, reliability, and responsiveness, for the comprehensive evaluation of symptoms during COPD management [1,10,11,12]. Although several clinical trials have proven that GLY could improve health-related quality of life, as measured by St. George’s Respiratory Questionnaire, little is known about whether GLY could be effective in improving health status for patients with COPD under routine clinical practice, particularly when evaluated using the CCQ [5,6,13].

We hypothesized that GLY used as monotherapy, or as part of combination treatment, during routine clinical care of COPD patients may improve health-related quality of life. Therefore, the primary purpose of this study was to evaluate the effectiveness of GLY on the health status of patients with COPD as measured by the CCQ in a routine clinical care setting. Our study was also aimed at assessing the safety and tolerability of GLY observed in clinical practice.

## 2. Materials and Methods

### 2.1. Study Design and Population

This multi-center, prospective, observational study was conducted between December 2014 and June 2015 at the Changhua Christian Hospital, the China Medical University Hospital, and the Taichung Veterans General Hospital, three medical centers in central Taiwan. Patients were enrolled if they had a physician-diagnosed COPD and the hospital clinician independently chose to prescribe GLY (Seebri^®^ Breezhaler^®^ capsules; Novartis Pharma AG, Basel, Switzerland), either with or without additional maintenance pharmacological therapies, prior to study enrollment. Patients were excluded if they were participating in an investigational drug trial or had COPD exacerbations at study enrollment The Institutional Review Boards and Ethics Committees at each participating hospital approved this study (approval number: SF14271A); all methods were performed in accordance with the Declaration of Helsinki. Informed consent was obtained from all participants.

### 2.2. Data Source and Collection

Clinical data, including demographics, smoking history, COPD history and medications, and comorbidities, were collected from electronic medical records; no specific visits, exams, or procedures were required. Each enrolled subject was followed for 6 months (± 4 weeks) or until study discontinuation. Outcome assessments were performed at the study enrollment and end of the study.

### 2.3. Patient Datasets/Subgroups Definition

Three datasets were considered in the analysis of the primary endpoint: the “Full Analysis Set (FAS)” included all subjects who provided informed consent and were prescribed with GLY; “Completers” were defined as participants who received the fixed COPD maintenance treatment prescribed throughout the 6-month study period and completed the study; and “Changers” were defined as those who changed their COPD maintenance treatment at any point during the study.

Furthermore, we categorized the participants at study enrollment into three subgroups: treatment-naïve patients (patients who had not received any COPD treatment before the study), add-on patients (patients who received GLY as a supplemental therapy) and switched patients (patients whose previous COPD medication was replaced with GLY either alone or as part of combination therapy).

### 2.4. Study Endpoints

To assess the effectiveness of GLY on health status, we calculated the mean change in CCQ total and domain scores between the study enrollment and the end of the study for the participants. Participants were asked to complete the CCQ (CCQ 7-day recall) upon study enrollment and at the end of the study. Briefly, the CCQ questionnaire consists of 10 items divided into three domains: symptoms, functional state, and mental state. It utilizes a 7-point scale where 0 indicates asymptomatic or no limitations and 6 indicates extremely symptomatic or completely limited. The CCQ has a minimal clinically important difference of 0.4 points [11,14].

To assess the safety and tolerability of GLY, adverse events and serious adverse events reported by both physicians and patients were monitored and recorded. Reasons for study discontinuation were also recorded.

### 2.5. Sample Size Calculation

To estimate the total sample size, studies that reported mean changes in the CCQ total score were identified in the literature, and the variability of the primary endpoint was considered in the sample size calculation. The mean changes in the CCQ total score from baseline reported by Reda AA et al. and Tashkin DP et al. were −0.54 ± 0.50 at week 26 and −0.52 ± 0.93 at week 24, respectively [14,15].

As the study aim was descriptive, the total sample size was calculated on the basis of the width of a two-sided 95% confidence interval (CI) from the mean change in the CCQ total score. To achieve the appropriate level of precision, a 95% CI width of 0.3 (± 0.15), a total of 100 evaluable subjects were required. Assuming a dropout rate of 10%, our goal was to enroll 110 patients.

### 2.6. Statistical Analysis

All statistical analyses were generated using SAS software version 9.4 (SAS Institute, Inc., Cary, NC, USA). Qualitative variables were summarized using descriptive statistics and reported as frequencies and percentages. Quantitative variables were summarized and reported as the mean, standard deviation, median, and minimum–maximum and compared with the paired sample t-test or the Wilcoxon signed-rank test between the study enrollment and the end of the study based on the normality assumption. The two-sided significance level was set at 0.05.

## 3. Results

### 3.1. Baseline Information

**Figure 1:**
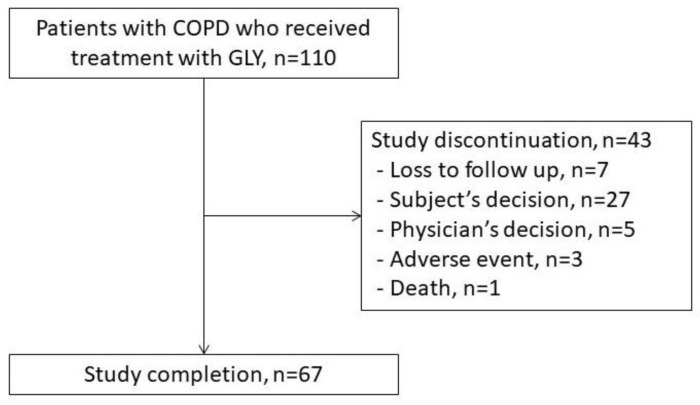
The patient enrolment flow chart. Abbreviations: COPD, chronic obstructive pulmonary disease; GLY, glycopyrronium.

Figure 1 shows the patient recruitment diagram. A total of 110 patients with COPD were eligible and enrolled in this study. Of the 110 participants, 67 subjects completed the study; 43 patients discontinued the study.

The majority of the participants were elderly men, and only Asian patients were enrolled in this study (Table 1). Most of the study population consisted of ex- or current smokers; around a quarter of the participants had moderate-to-severe exacerbations in the 12 months before enrollment; seven (6.36%) enrollees were also diagnosed of having asthma (Table 1).

### 3.2. Change in CCQ Total and Domain Scores

**Figure 2:**
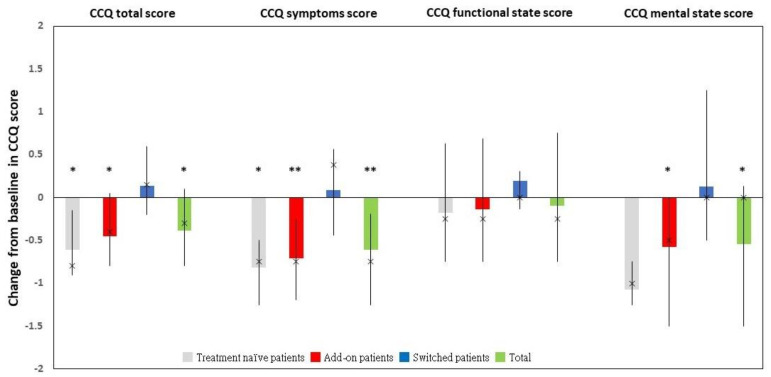
The change from baseline in CCQ total and domain scores in the full analysis dataset and its corresponding subgroups. * *p* < 0.05. ** *p* < 0.001. Abbreviations: CCQ, clinical COPD questionnaire.

Because of one add-on patient’s death and seven add-on patients’ lost follow-up during the study period, the statistical comparison of the CCQ scores between the enrollment and end of the study was completed for 102 enrollees. Following treatment with GLY, the FAS had a statistically significant improvement in the CCQ total, symptoms, and mental state scores (Figure 2). There was no significant improvement in the functional state score. The details were shown in Table S1 in the Supplementary Materials.

Among the FAS (*n* = 102), the CCQ total and symptoms scores were significantly lower for treatment-naïve patients at study completion when compared with those at enrollment. Add-on patients had significant improvements in the CCQ total, symptoms, and mental state scores. However, switched patients exhibited no significant change in either the CCQ total score or any other CCQ domain score (Figure 2). The detailed information was available in Table S1 in the Supplementary Materials.

**Figure 3:**
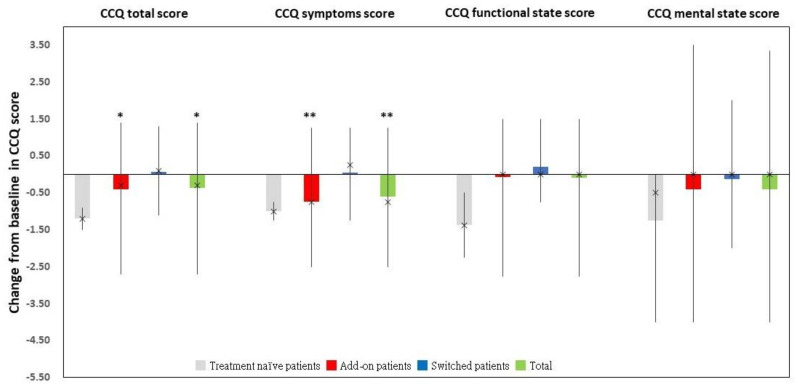
The change from baseline in CCQ total and domain scores in the “Completers” dataset and its corresponding subgroups. * *p* < 0.05. ** *p* < 0.001. Abbreviations: CCQ, clinical COPD questionnaire.

**Figure 4:**
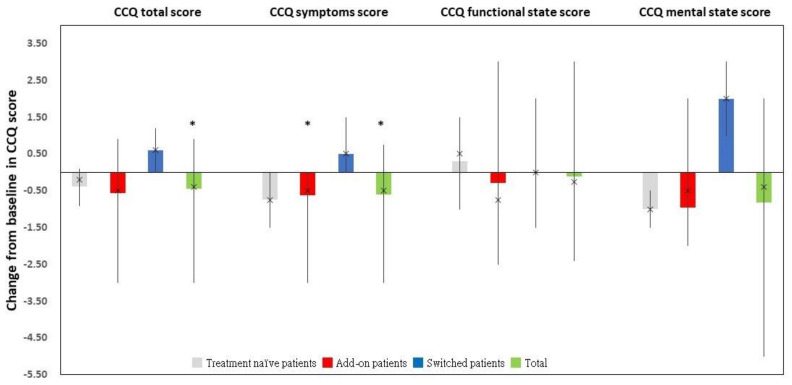
The change from baseline in CCQ total and domain scores in the “Changers” dataset and its corresponding subgroups. * *p* < 0.05. Abbreviations: CCQ, clinical COPD questionnaire.

For the 45 “Completers” (Figure 3) and 33 “Changers” (Figure 4), the CCQ total and symptoms scores were significantly lower at the end of the study when compared to those at baseline. The functional state and mental state scores insignificantly changed from the baseline. The details were provided in Tables S2 and S3 in the Supplementary Materials.

Among the “Completers” (*n* = 45), add-on patients exhibited a significant improvement in CCQ total and symptom scores between the end of the study and baseline (Figure 3 and Table S2 in the Supplementary Materials). Meanwhile, compared to that at enrollment, add-on patients had a significantly decreased CCQ symptoms score at the end of the study among the “Changers” (*n* = 33) (Figure 4 and Table S3 in the Supplementary Materials).

### 3.3. Safety

Adverse events were reported by 52.73% of the FAS during the 6-month observational period (Table 2). The most common adverse event was the worsening of COPD. Only one adverse event (dizziness) was suspected to be related to the study treatment. Three patients (2.73%) discontinued the study because of an adverse event such as a diagnosis of small cell lung cancer (*n* = 1), dizziness (*n* = 1), and pneumonia (*n* = 1) followed by no more use of the study drug (GLY). One patient died during the observational period from a cerebral infarction that was not suspected to be related to the study drug (Figure 1 and Table 2).

## 4. Discussion

GLARE-Taiwan, the first real-world, observational study assessing the effectiveness and tolerability of GLY in a Taiwanese population with COPD, reflects the clinical implications of GLY as the maintenance pharmacological therapy for COPD in clinical practice. In this study, GLY significantly improved health status as determined by CCQ scores in all three datasets of FAS, “Completers”, and “Changers”, particularly in treatment naïve and add-on patients. Additionally, 52.73% of study subjects reported adverse events in the 6-month follow-up period while 2.73% of participants discontinued the study and GLY treatment because of adverse events.

Consistent with the findings that GLY provided significant improvements in health status as determined by St George’s Respiratory Questionnaire for moderate-to-severe COPD patients in the GLOW1, GLOW2, and GLOW7 phase III randomized controlled trials (GLY versus placebo and open-label tiotropium), and the GEM1 (Glycopyrrolate Effect on syMptoms and lung function) clinical study [5,6,13,16], we found that treatment with GLY significantly improved health status as measured by CCQ for COPD patients in all the studied datasets and certain subgroups of the dataset in a real-world setting. This indicates that GLY could consistently improve health-related quality of life in patients with COPD regardless of the setting, dataset, prescription timing, or questionnaire used to evaluate health status although switched patients did not show significant improvements in CCQ scores in the present study, likely because GLY, basically, was used only as a replacement for the existed maintenance pharmacological treatment in such study subgroup.

We found that 52.73% (*n* = 58) of the participants reported adverse events following GLY treatment during the 6-month observational period. This was similar to that of the previous study with an adverse event frequency of 57.5% in patients with COPD who received GLY treatment during the 26-week follow-up in the GLOW1 trial [5]. Furthermore, several reports showed the incidence of adverse events after treatment with GLY for COPD as follows: 76.6% at week 52 in GLOW2, 29.1% at week 3 in GLOW3, 40.4% at week 12 in GLOW5, 43.6% at week 12 in GLOW7, and 42.8% at week 12 in GEM1 [6,7,8,13,16]. Meanwhile, the proportion of study drug discontinuation due to adverse events was 2.73% (*n* = 3), which was similar to that of 2.6% reported by Wang C et al. and relatively low compared to those reported by D’Urzo A et al. (5.8%) and Kerwin E et al. (8.0%) [5,6,13]. Collectively, these data suggest that GLY is a safe and tolerable treatment option for COPD.

A strength of this study is that only two interviews were needed to complete the CCQ for the participant with a high intraclass correlation coefficient of 0.869 and the study subject did not require any specific visit, exam, or procedure. Another strength is that all medications, including GLY, were supplied to patients with a reimbursement from the Taiwan National Health Insurance and did not require an extra personal cost. Additionally, the patients with a history of asthma were not excluded from this study. Taken together, this further confirmed the practicality of the study design, lessened the interference of the study results when applied in a clinical setting, and compensates for our study’s limitation as a non-interventional study. The lack of intervention meant the absence of a control group, which made solid comparisons impossible and resulted in a possible study effect of significant improvements in the CCQ total and domain scores. Moreover, all the participants were Asians, more than 90% of enrollees being male, the small number of enrolled patients, particularly those in the datasets of “Completers” and “Changers” and in the study subgroups of treatment-naïve, add-on, and switched patients, and absence of exacerbation history in the previous 12 months in 73.39% of the enrollees limit the statistical power and generalizability of our findings.

Several clinical trials have clarified that GLY could improve health status, lung function, and dyspnea, delay time to clinically important deteriorations, and reduce exacerbations for patients with COPD with a superior and faster bronchodilation when compared to tiotropium [5,6,7,13,16,17,18]. In addition, GLY has shown a similar safety profile as compared to placebo [5,6,13]. Together with our findings that GLY could improve the health status of COPD patients, and was shown to have good safety and tolerability profile in a real-world practice, GLY may be a better choice for LAMA in the management of COPD. A large-scale, well-designed, real-world study should be implemented to validate our findings in the future.

## 5. Conclusions

This study found that GLY could improve health status and had a good safety and tolerability profile for patients with COPD in a clinical setting, and provides evidence that GLY could be an optimal LAMA when treating COPD patients. These findings should be taken into consideration when making treatment decisions in routine clinical practice.

## Figures and Tables

**Table 1:** Baseline information of the enrolled participants.

Characteristics	Total (*n* = 110)
**Age (years)**	
Mean ± SD	71.25 ± 10.60
Median	71.55
Min-Max	34–95
<40 years	2 (1.82%)
40–64 years	25 (22.73%)
65–74 years	38 (34.55%)
≥75 years	45 (40.91%)
**Male**	101 (91.82)
**Asian**	110 (100%)
**BMI (kg/m^2^)**	
Mean ± SD	24.70 ± 4.38
Median	24.40
Min-Max	14.50–40.70
**Smoking status**	
Non-smoker	13 (11.82%)
Ex-smoker	68 (61.82%)
Current smoker	29 (26.36%)
**Airflow limitation**	
GOLD 1	7 (6.36%)
GOLD 2	53 (48.18%)
GOLD 3	41 (37.27%)
GOLD 4	9 (8.18%)
**COPD duration (years)**	
Mean ± SD	3.84 ± 4.17
**History of moderate-to-severe COPD exacerbations within one year**	
Mean ± SD	0.44 ± 0.92
0	80 (73.39%)
1	19 (17.43%)
2	5 (4.59%)
3	1 (0.92%)
4	4 (3.67%)
Missing	1 (0.91%)
**History of severe exacerbations within one year**	
Mean ± SD	0.22 ± 0.58
0	91 (83.49%)
1	14 (12.84%)
2	3 (2.75%)
4	1 (0.92%)
Missing	1 (0.92%)
**Inhaled COPD maintenance medication**	
None	9 (8.18%)
LAMA alone	35 (31.82%)
Tiotropium	35 (31.82%)
LABA alone	49 (44.55%)
Indacaterol	36 (32.73%)
Salmeterol	13 (11.82%)
ICS/LABA	17 (15.45%)
Salmeterol/Fluticasone	9 (8.18%)
Budesonide/Formoterol	6 (5.45%)
Formoterol/Beclomethasone	2 (1.82%)
**Oral COPD maintenance medication**	
Prednisolone	4 (3.64%)
Methylxanthines	53 (48.18%)
Roflumilast	1 (0.91%)
**Subgroup**	
Treatment-naïve	9 (8.18%)
Add-on	75 (68.18%)
Switched	26 (23.64%)
**Comorbidity**	
Any	37 (33.64%)
Hypertension	16 (14.55%)
Benign prostatic hyperplasia	13 (11.82%)
Allergic rhinitis	10 (9.09%)
Diabetes mellitus	10 (9.09%)
Coronary artery disease	9 (8.18%)
Asthma	7 (6.36%)
Gastroesophageal reflux disease	7 (6.36%)
Chronic renal failure	6 (5.45%)
Hyperlipidemia	6 (5.45%)

Abbreviations: BMI, body mass index; COPD, chronic obstructive pulmonary disease; GOLD, The Global Initiative for Chronic Obstructive Lung Disease; ICS, inhaled corticosteroid; LABA, long-acting β2-agonist; LAMA, long-acting muscarinic antagonist; Max, maximum; Min, minimum; SD, standard deviation.

**Table 2:** Adverse events of the Full Analysis Set.

	Total (*n* = 110)
**Patients with any AE**	58 (52.73%)
**Common AEs (occurring in >3.0% of participants)**	
COPD worsening	9 (8.18%)
Constipation	5 (4.55%)
Dizziness	5 (4.55%)
Edema	5 (4.55%)
Urinary tract infection	5 (4.55%)
Dyspepsia	4 (3.64%)
Upper respiratory tract infection	4 (3.64%)
**Death**	1 (0.90%)
**Discontinuation due to AE**	3 (2.73%)

Abbreviations: AE, adverse event; COPD, chronic obstructive pulmonary disease.

## Data Availability

Data is contained within the article.

## Supplementary Materials

The following supporting information can be downloaded at: https://www.mdpi.com/article/10.3390/jcm11206210/s1, Table S1: The change in CCQ total and domain scores between the baseline and end of study in the full analysis dataset and its corresponding subgroups; Table S2: The change in CCQ total and domain scores between the baseline and end of study in the “Completers” dataset and its corresponding subgroups; Table S3: The change in CCQ total and domain scores between the baseline and end of study in the “Changers” dataset and its corresponding subgroups.
