# Supplementary material for: The Effectiveness and Tolerability of Glycopyrronium for Patients with Chronic Obstructive Pulmonary Disease in a Clinical Setting: GLARE-Taiwan
Source: J Clin Med. 2022 Oct 21;11(20):6210. doi: 10.3390/jcm11206210 (PMC9604569; doi:10.3390/jcm11206210)
Supplement: Supplementary file 1 [file jcm-11-06210-s001.zip › jcm-1977145-supplementary.pdf]

Journal of
Clinical Medicine

MDPI

Article

The effectiveness and tolerability of glycopyrronium for patients with chronic obstructive pulmonary disease in a clinical setting: GLARE-Taiwan

Wei-Chang Huang [1,2,3,4,5,†], Sheng-Hao Lin [2,6,†], Liang-Wen Hang [7,*], Ching-Hsiung Lin [2,6,*] and Jeng-Yuan Hsu [1,8,*]

1 Division of Chest Medicine, Department of Internal Medicine, Taichung Veterans General Hospital, Taichung, 407, Taiwan
2 Department of Post-Baccalaureate Medicine, College of Medicine, National Chung Hsing University, Taichung, 402, Taiwan
3 Ph.D. Program in Translational Medicine, National Chung Hsing University, Taichung, 402, Taiwan;
4 School of Medicine, Chung Shan Medical University, Taichung, 402, Taiwan
5 Department of Medical Technology, Jen-Teh Junior College of Medicine, Nursing and Management, Miaoli, 350, Taiwan
6 Division of Chest Medicine, Department of Internal Medicine, Changhua Christian Hospital, Changhua, Taiwan
7 Sleep Medicine Center, Department of Pulmonary and Critical Care Medicine, China Medical University Hospital, Taichung, Taiwan
8 School of Physical Therapy, Chung Shan Medical University, Taichung, 402, Taiwan;
* Correspondence: huchen0328@gmail.com (J.-Y.H.); teddy@cch.org.tw (C.-H.L.) and lungwen.hang@gmail.com (L.-W.H.).
† These authors contributed equally to this work.

Citation: Huang, W.-C.; Lin, S.-H.; Hang, L.-W.; Lin, C.-H.; Hsu, J.-Y. The effectiveness and tolerability of glycopyrronium for patients with chronic obstructive pulmonary disease in a clinical setting: GLARE-Taiwan. *J. Clin. Med.* **2022**, *11*, x. https://doi.org/10.3390/xxxxx

Academic Editor(s):

Received: date
Accepted: date
Published: date

Publisher's Note: MDPI stays neutral with regard to jurisdictional claims in published maps and institutional affiliations.

Copyright: © 2022 by the authors. Submitted for possible open access publication under the terms and conditions of the Creative Commons Attribution (CC BY) license (http://creativecommons.org/licenses/by/4.0/).

Table S1. The change in CCQ total and domain scores between the baseline and end of study in the full analysis dataset and its corresponding subgroups.

	Treatment naïve patients (n=9)	Add-on patients (n=67)	Switched patients (n=26)	Total (n=102)
**CCQ total score**				
Baseline				
Mean±SD	1.80±0.86	2.18±1.05	1.52±1.07	2.02±1.04
Median (Min-Max)	1.85 (1.1, 2.33)	2.00 (1.43, 2.90)	1.20 (1.00, 1.80)	1.80 (1.3, 2.7)
Month 6/EOS				
Mean±SD	1.4 ±0.96	1.69±1.19	1.45±1.10	1.63±1.14
Median (Min-Max)	1.10 (0.75, 2.15)	1.35 (0.80, 2.35)	1.20 (0.70, 1.75)	1.30 (0.80, 2.25)
Change from baseline				
Mean±SD	-0.61±0.57	-0.45±0.95	0.14±0.73	-0.39±0.90
Median (Min-Max)	-0.80 (-0.90, -0.15)	-0.40 (-0.80, 0.05)	0.15 (-0.20, 0.60)	-0.30 (-0.80, 0.10)
P-value	0.047*	0.004*	0.484	0.002*
**CCQ symptoms score**				
Baseline				
Mean±SD	2.11±1.04	2.46±1.05	1.75±1.05	2.30±1.07
Median (Min-Max)	1.88 (1.50, 2.69)	2.25 (1.75, 3.25)	1.50 (1.00, 2.00)	2.00 (1.50, 3.06)
Month 6/EOS				
Mean±SD	1.46±0.60	1.77±1.25	1.53±0.86	1.70±1.14
Median (Min-Max)	1.25 (1.00, 2.00)	1.50 (0.81, 2.50)	1.38 (0.94, 1.94)	1.50 (1.00, 2.50)
Change from baseline				
Mean±SD	-0.82±0.55	-0.71±0.90	0.09±0.87	-0.61±0.90
Median (Min-Max)	-0.75 (-1.25, -0.50)	-0.75 (-1.19, -0.25)	0.38 (-0.44, 0.56)	-0.75 (-1.25, -0.19)
P-value	0.031*	<0.001**	0.719	<0.001**
**CCQ functional state score**				
Baseline				
Mean±SD	1.38±1.02	1.92±1.27	1.38±1.09	1.75±1.22
Median (Min-Max)	1.13 (0.50, 2.00)	1.75 (1.00, 2.69)	1.00 (0.75, 1.50)	1.50 (1.00, 2.50)
Month 6/EOS				
Mean±SD	1.36±1.46	1.79±1.39	1.41±1.22	1.68±1.37
Median (Min-Max)	1.00 (0.75, 1.25)	1.50 (1.00, 2.19)	1.00 (0.69, 1.94)	1.50 (0.75, 2.06)
Change from baseline				
Mean±SD	-0.18±1.24	-0.14±1.21	0.19±0.79	-0.10±1.15
Median (Min-Max)	-0.25 (-0.75, 0.63)	-0.25 (-0.75, 0.69)	0.00 (-0.13, 0.31)	-0.25 (-0.75, 0.75)
P-value	0.844	0.466	0.625	0.529
**CCQ mental state score**				
Baseline				
Mean±SD	2.05±1.76	2.12±1.92	1.35±1.68	2.01±1.87
Median (Min-Max)	1.75 (1.00, 2.50)	1.50 (0.13, 3.88)	0.50 (0.00, 2.50)	1.50 (0.50, 3.13)
Month 6/EOS				
Mean±SD	1.57±1.92	1.32±1.69	1.38±1.62	1.36±1.68
Median (Min-Max)	1.00 (0.00, 2.75)	0.75 (0.00, 2.00)	0.75 (0.00, 2.38)	1.00 (0.00, 2.00)
Change from baseline				
Mean±SD	-1.07±1.62	-0.58±1.77	0.13±1.55	-0.54±1.72
Median (Min-Max)	-1.00 (-1.25, -0.75)	-0.50 (-1.50, 0.00)	0.00 (-0.50, 1.25)	0.00 (-1.50, 0.13)
P-value	0.156	0.038*	>0.99	0.021*

*p<0.05, **p<0.001. Abbreviations: CCQ, clinical COPD questionnaire; EOS, end of study; Max, maximum; Min, minimum; SD, standard deviation.

Table S2. The change in CCQ total and domain scores between the baseline and end of study in the "Completers" dataset and its corresponding subgroups.

	Treatment naïve patients (n=4)	Add-on patients (n=34)	Switched patients (n=7)	Total (n=45)
**CCQ total score**				
Baseline				
Mean±SD	2.23±0.71	2.09±0.96	1.19±0.60	1.96±0.95
Median (Min-Max)	2.10 (1.60, 3.00)	1.90 (0.40, 4.00)	1.10 (0.30, 2.10)	1.80 (0.30, 4.4)
Month 6/EOS				
Mean±SD	1.35±1.06	1.67±1.18	1.26±1.03	1.58±1.13
Median (Min-Max)	1.35 (0.60, 2.10)	1.20 (0.20, 4.30)	1.10 (0.20, 3.40)	1.20 (0.20, 4.30)
Change from baseline				
Mean±SD	-1.20±0.42	-0.41±0.95	0.07±0.77	-0.36±0.93
Median (Min-Max)	-1.20 (-1.50, -0.90)	-0.30 (-2.70, 1.40)	0.10 (-1.10, 1.30)	-0.30 (-2.70, 1.40)
P-value	0.500	0.029*	0.719	0.022*
**CCQ symptoms score**				
Baseline				
Mean±SD	2.25±0.50	2.49±0.97	1.36±0.56	2.30±0.98
Median (Min-Max)	2.25 (1.75, 2.75)	2.25 (0.25, 4.75)	1.00 (0.74, 2.25)	2.25 (0.25, 4.75)
Month 6/EOS				
Mean±SD	1.50±0.71	1.77±1.33	1.39±0.83	1.68±1.22
Median (Min-Max)	1.50 (1.00, 2.00)	1.50 (0.00, 5.00)	1.25 (0.50, 3.00)	1.50 (0.00, 5.00)
Change from baseline				
Mean±SD	-1.00±0.35	-0.74±0.91	0.04±0.92	-0.61±0.93
Median (Min-Max)	-1.00 (-1.25, -0.75)	-0.75 (-2.50, 1.25)	0.25 (-1.25, 1.25)	-0.75 (-2.50, 1.25)
P-value	0.500	<0.001**	0.906	<0.001**
**CCQ functional state score**				
Baseline				
Mean±SD	1.83±1.44	1.8±1.14	1.03±0.73	1.74±1.12
Median (Min-Max)	1.00 (1.00, 3.40)	1.75 (0.00, 4.25)	1.00 (0.00, 2.25)	1.50 (0.00, 4.25)
Month 6/EOS				
Mean±SD	0.88±0.53	1.84±1.26	1.25±1.22	1.68±1.24
Median (Min-Max)	0.88 (0.50, 1.25)	1.50 (0.50, 5.50)	1.00 (0.00, 3.75)	1.50 (0.00, 5.50)
Change from baseline				
Mean±SD	-1.38±1.24	-0.07±1.03	0.21±0.85	-0.09±1.03
Median (Min-Max)	-1.38 (-2.25, -0.50)	0.00 (-2.75, 1.50)	0.00 (-0.75, 1.50)	0.00 (-2.75, 1.50)
P-value	0.500	0.721	0.625	0.613
**CCQ mental state score**				
Baseline				
Mean±SD	3.00±0.87	1.70±1.82	1.14±1.44	1.70±1.75
Median (Min-Max)	2.50 (2.50, 4.00)	1.00 (0.00, 6.00)	0.00 (0.00, 3.00)	1.50 (0.00, 6.00)
Month 6/EOS				
Mean±SD	2.00±2.82	1.12±1.53	1.00±1.32	1.15±1.52
Median (Min-Max)	2.00 (0.00, 4.00)	0.50 (0.00, 6.00)	0.50 (0.00, 3.50)	0.50 (0.00, 6.00)
Change from baseline				
Mean±SD	-1.25±3.89	-0.41±1.68	-0.14±1.46	-0.41±1.72
Median (Min-Max)	-0.50 (-4.00, 0.00)	0.00 (-4.00, 3.50)	0.00 (-2.0, 2.0)	0.00 (-4.00, 3.50)
P-value	0.954	0.197	0.998	0.152

*p<0.05, **p<0.001. Abbreviations: CCQ, clinical COPD questionnaire; EOS, end of study; Max, maximum; Min, minimum; SD, standard deviation.

Table S3. The change in CCQ total and domain scores between the baseline and end of study in the "Changers" dataset and its corresponding subgroups.

	Treatment naïve patients (n=4)	Add-on patients (n=19)	Switched patients (n=10)	Total (n=33)
		**CCQ total score**		
Baseline				
Mean±SD	2.10±0.85	2.36±1.02	1.33±0.78	2.18±0.97
Median (Min-Max)	2.00 (1.00, 3.50)	2.20 (0.90, 4.50)	1.10 (0.70, 2.20)	2.00 (0.70, 4.50)
		Month 6/EOS		
Mean±SD	1.48±1.05	1.74±1.26	2.80±0.00	1.73±1.18
Median (Min-Max)	1.10 (0.30, 2.90)	1.40 (0.20, 4.20)	2.80 (2.80, 2.80)	1.40 (0.20, 4.2)
		Change from baseline		
Mean±SD	-0.38±0.44	-0.56±0.98	0.60±0.00	-0.45±0.87
Median (Min-Max)	-0.20 (-0.90, 0.10)	-0.50 (-3.00, 0.90)	0.60 (0.00, 1.20)	-0.40 (-3.00, 0.90)
P-value	0.188	0.055	0.622	0.036*
		**CCQ symptoms score**		
Baseline				
Mean±SD	2.48±1.09	2.43±0.98	1.42±0.63	2.35±1.02
Median (Min-Max)	2.25 (1.25, 4.50)	2.50 (0.75, 4.50)	1.50 (0.75, 2.00)	2.13 (0.75, 4.50)
		Month 6/EOS		
Mean±SD	1.45±0.65	1.79±1.12	2.50±0.00	1.74±0.99
Median (Min-Max)	1.25 (0.75, 2.25)	2.00 (0.25, 4.25)	2.50 (2.50, 2.50)	2.00 (0.25, 4.25)
		Change from baseline		
Mean±SD	-0.75±0.64	-0.63±0.91	0.50±0.00	-0.61±0.85
Median (Min-Max)	-0.75 (-1.50, 0.00)	-0.50 (-3.00, 0.00)	0.50 (0.00, 1.50)	-0.50 (-3.00, 0.75)
P-value	0.125	0.019*	0.632	0.006*
		**CCQ functional state score**		
Baseline				
Mean±SD	1.52±0.96	2.10±1.19	1.50±0.87	1.84±1.10
Median (Min-Max)	1.25 (0.25, 3.00)	1.88 (0.75, 5.50)	1.00 (1.00, 2.50)	1.75 (0.25, 5.50)
		Month 6/EOS		
Mean±SD	1.55±1.72	1.67±1.71	2.50±0.00	1.68±1.63
Median (Min-Max)	1.00 (0.00, 4.50)	1.50 (0.00, 5.25)	2.50 (2.50, 2.50)	1.25 (0.00, 5.25)
		Change from baseline		
Mean±SD	0.30±0.96	-0.29±1.57	0.00±0.00	-0.12±1.38
Median (Min-Max)	0.50 (-1.00, 1.50)	-0.75 (-2.5, 3.00)	0.00 (-1.50, 2.00)	-0.25 (-2.50, 3.00)
P-value	0.625	0.486	0.988	0.713
		**CCQ mental state score**		
Baseline				
Mean±SD	2.50±2.18	2.75±2.04	0.83±1.04	2.48±2.05
Median (Min-Max)	2.00 (0.00, 6.00)	2.50 (0.00, 5.50)	0.50 (0.00, 2.00)	2.00 (0.00, 6.00)
		Month 6/EOS		
Mean±SD	1.40±1.85	1.77±2.00	4.00±0.00	1.79±1.94
Median (Min-Max)	1.00 (0.00, 4.50)	1.00 (0.00, 6.00)	4.00 (4.00, 4.00)	1.40 (0.00, 6.00)
		Change from baseline		
Mean±SD	-1.00±0.35	-0.96±1.95	2.00±0.00	-0.82±1.74
Median (Min-Max)	-1.00 (-1.50, -0.5)	-0.50 (-2.00, 2.00)	2.00 (1.00, 3.00)	-0.40 (-5.00, 2.00)
P-value	0.063	0.100	0.455	0.056

*p<0.05. Abbreviations: CCQ, clinical COPD questionnaire; EOS, end of study; Max, maximum; Min, minimum; SD, standard deviation.
